# Four-Step Framework for Valve-in-Valve Transcatheter Aortic Valve Replacement for Failed Surgical and Transcatheter Aortic Bioprostheses

**DOI:** 10.31083/RCM43142

**Published:** 2025-09-29

**Authors:** Milos Brankovic, Hassan Akram, Aisha Shabbir, Laith Alhuneafat, Abhishek Sharma

**Affiliations:** ^1^Cardiovascular Division, Department of Medicine, University of Minnesota, Minneapolis, MN 55455, USA; ^2^Department of Medicine, University of Minnesota, Minneapolis, MN 55455, USA; ^3^Division of Cardiology, Department of Medicine, Rutgers New Jersey Medical School, Newark, NJ 07103, USA

**Keywords:** valve-in-valve, ViV, transcatheter aortic valve replacement, TAVR, transcatheter aortic valve implantation, TAVI, structural valve deterioration, bioprosthetic valve failure, transcatheter aortic valve-in-surigical aortic valve, transcatheter aortic valve-in-transcatheter aortic valve

## Abstract

Transcatheter aortic valve replacement (TAVR), originally developed to treat native aortic valve disease, has become increasingly adopted in the valve-in-valve (ViV) setting to manage bioprosthetic valve dysfunction of both surgically implanted bioprostheses (TAV-in-SAV) and prior transcatheter valves (TAV-in-TAV). Recent advances have significantly refined the ViV technique to address procedural challenges, including suboptimal hemodynamic outcomes and the risk of coronary obstruction. This review summarizes the current clinical data supporting the use of TAVR in a ViV setting and outlines a structured, four-step framework that encompasses procedural planning, including determining the perioperative risk for a patient, identifying the mode of valve failure, recognizing valve-specific implantation strategies, and assessing concomitant structural lesions. This review also aims to synthesize current evidence into a clinically actionable format, helping to guide heart team discussions, pre-procedural planning, and patient counseling.

## 1. Introduction

Since its first-in-human application, transcatheter aortic valve replacement (TAVR) has evolved into a cornerstone therapy for severe aortic stenosis. The U.S. Food and Drug Administration granted initial approval in 2012 for patients at high or prohibitive surgical risk, followed by expanded indications for intermediate-risk patients in 2016 and low-risk patients in 2019 [1]. The TAVR has become one of the most commonly performed aortic valve procedures in the United States, exceeding surgical aortic valve replacement in 2019 [2].

While TAVR was originally developed to treat native aortic valve stenosis, TAVR technology has quickly expanded to the valve-in-valve (ViV) setting to address bioprosthetic valve dysfunction of previously implanted surgical (TAV-in-SAV) and transcatheter valves (TAV-in-TAV). The advantage of avoiding lifelong anticoagulation and the inherent limited durability of bioprosthetic valves have also contributed to a growing preference for ViV TAVR even among younger patients. In 2019 alone, more than 4500 ViV TAVR procedures were performed in the United States. While TAV-in-SAV currently comprises the majority of ViV procedures, the expanding use of TAVR in younger and lower-risk populations is projected to shift this balance. By 2028, TAV-in-TAV is expected to become the predominant ViV approach. Overall, the total annual volume of ViV TAVR is projected to reach approximately 42,000 cases in the U.S. by 2035 [3].

Given the rise of ViV TAVR procedures, this review article proposes a structured, four-step framework that encompasses ViV planning for failed surgical and transcatheter aortic bioprostheses. This includes determination of (1) the patient’s perioperative risk, anticipated life expectancy, and preference, (2) index-valve mode of failure, (3) valve-specific implantation strategies, and (4) assessment of concomitant structural lesions. Our aim was to summarize the latest literature data, guideline-based recommendations, and procedural considerations in a stepwise format that can be applied in a real-world clinical workflow.

## 2. Current Guideline Recommendations for Failed Surgical and Transcatheter Aortic Bioprostheses

The 2020 American College of Cardiology/American Heart Association (ACC/AHA) and the 2021 European Society of Cardiology/European Association for Cardiac and Thoracic Surgery (ESC/EACTS) guidelines have endorsed redo surgical aortic valve replacement (SAVR) in symptomatic patients with severe stenosis of bioprosthetic aortic valve and low to intermediate surgical risk (class 1 recommendation) [4, 5]. In patients with high or prohibitive surgical risk, American and European guidelines provide a class 2a recommendation for transfemoral ViV TAVR in those with suitable anatomy. In cases with severe prosthetic valvular or paravalvular regurgitation causing heart failure symptoms or hemolysis, both guidelines endorse redo SAVR as a class 1 recommendation for low to intermediate surgical risk patients. A redo SAVR can be considered in asymptomatic patients with severe regurgitation and low surgical risk (class 2a recommendations). Both guidelines considered the ViV TAVR or percutaneous leak closure as a reasonable option for high- and prohibitive-risk individuals with heart failure symptoms or hemolysis and suitable anatomy (class 2a).

Treatment of failed TAVR is currently an evolving area with limited specific guidelines for its management. There are currently no definitive recommendations regarding what type of transcatheter prosthesis should be implanted or how the procedure should be performed. The 2020 ACC/AHA and 2021 ESC/EACTS guidelines provide mostly general recommendations for managing bioprosthetic valve failure. Both guidelines stress the role of a multidisciplinary heart team and individualized evaluation to optimize outcomes in the setting of a failed TAVR valve. They are consistent about the comprehensive evaluation of factors such as age, comorbidities, anatomical suitability, and procedural risks. They also emphasize the importance of shared decision-making, ensuring that patients are well-informed about the potential risks and benefits of the redo intervention.

## 3. ViV TAVR in Failed Surgical Aortic Bioprostheses

### 3.1 Clinical Outcomes and Complications of TAV-in-SAV Studies

Current evidence primarily relies on observational studies without large randomized controlled trials (RCTs) directly comparing TAV-in-SAV and redo-SAVR. In a 2021 meta-analysis, Sá *et al*. [6] investigated observational studies published from 2015 to 2020 encompassing 16,207 patients with degenerated surgical aortic valves. The pooled analysis showed favorable 30-day all-cause mortality in patients treated with TAV-in-SAV compared to redo SAV (odds ratio [OR] and 95% confidence interval [CI]: 0.52 [0.39 to 0.68], *p* < 0.001). However, the authors also reported higher odds of severe post-procedural patient-prosthesis mismatch (PPM) in the TAV-in-SAV group compared to the redo SAVR group (OR [95% CI]: 4.63 [3.05 to 7.03], *p* < 0.001). However, the TAV-in-SAV group had lower odds of major bleeding (OR: 0.48 [0.28 to 0.80], *p* = 0.013) and shorter length of stay (absolute difference: –3.30 days [–4.52 to –2.08], *p* < 0.001) compared to the redo SAVR group. No difference was observed in the rates of stroke, myocardial infarction, permanent pacemaker implantation, paravalvular leak (PVL), renal failure, and major vascular complications during a short-term follow-up.

Subsequently, Raschpichler *et al*. [7] expanded a meta-analysis to mid-term outcomes in patients undergoing TAV-in-SAV versus redo SAVR. The authors also demonstrated lower 30-day mortality in patients treated with TAV-in-SAV compared to redo SAVR (2.8% vs. 5.0%; risk ratio [RR] and [95% CI]: 0.55 [0.34 to 0.91], *p* = 0.02). However, no difference was found in two-year mortality rates (hazard ratio [HR] and [95% CI]: 1.27 [0.72 to 2.2], *p* = 0.37). They also showed a higher chance of prosthetic regurgitation (RR [95% CI]: 4.18 [1.88 to 9.3], *p* = 0.003) and severe PPM (3.12 [2.35 to 4.1]) in patients treated with TAV-in-SAV compared to redo SAVR. No significant difference was observed between groups for stroke, myocardial infarction, or permanent pacemaker implantation during a short-term follow-up.

In a 2023 meta-analysis, Formica *et al*. [8] confirmed a higher 30-day all-cause mortality in patients who underwent redo SAVR compared to TAV-in-SAV (HR [95% CI]: 2.12 [1.49 to 3.03], *p* < 0.0001). The cumulative incidence of all-cause mortality at one year was 12.9% (95% CI: 11.3% to 14.6%) in the redo SAVR group compared to 9.9% (95% CI: 8.5% to 11.5%) in the TAV-in-SAV group. At five years, the cumulative incidence of all-cause mortality was 25.4% (95% CI: 22.6% to 28.6%) in the redo SAVR group and 27.6% (95% CI: 24.9% to 30.1%) in the TAV-in-SAV group. However, among patients who survived beyond the first year, a landmark analysis showed that redo SAVR was associated with a significantly lower risk of mortality compared to TAV-in-SAV (HR: 0.59; 95% CI: 0.44 to 0.79; *p* < 0.001). These findings were consistent with the updated five-year meta-analysis by Sá *et al*. [9]. In their pooled analysis, the TAV-in-TAV group had lower 30-day mortality rates (OR [95% CI]: 0.52 [0.39 to 0.68], *p* < 0.001), less major bleeding (OR [95% CI]: 0.48 [0.28 to 0.80], *p* = 0.013), but higher rates of severe PPM (OR [95% CI]: 4.63 [3.05 to 7.03], *p* < 0.001). The authors also demonstrated that PPM became associated with all-cause mortality after eight months of follow-up. Their time-to-event analyses revealed that the relative risk between ViV TAVR and redo SAVR changes over time, indicating the presence of time-varying treatment effects.

These findings were recently reproduced by Awtry *et al*. [10], who investigated the five-year outcomes of Medicare beneficiaries who underwent either TAV-in-SAV or redo SAVR for a degenerated surgical aortic valve. In their propensity score–matched cohort analysis, patients who underwent redo SAVR had higher rates of 30-day mortality (9.6% vs. 5.0%, *p* < 0.001), permanent pacemaker implantation (11.7% vs. 6.9%, *p* < 0.001), and renal failure (12.9% vs. 3.6%, *p* < 0.001). However, their landmark analysis demonstrated a survival benefit of redo SAVR over TAV-in-SAV for patients who survived the first 20 months and remained stable at five years (62.3% vs. 46.7%, *p* < 0.001).

### 3.2 Patient-Reported Outcomes of TAV-in-SAV Studies

The Global Valve-in-Valve Registry represents the first large-scale assessment of the early experience with TAV-in-SAV in patients with degenerated surgical bioprostheses deemed unsuitable for redo surgery [11]. This registry demonstrated a marked improvement in functional status, with the proportion of patients in New York Heart Association (NYHA) class III or IV decreasing from 94% at baseline to 7.4% at 30 days post-procedure, which sustained through one-year follow-up. Similarly, other single-center studies demonstrated improved NYHA class following TAV-in-SAV [12, 13]. However, when compared directly, the magnitude of NYHA class improvement did not differ between patients undergoing TAV-in-SAV and those receiving redo SAVR.

The PARTNER 2 ViV registry data also showed improved NYHA functional class from baseline to 30 days and one year in high-risk patients who underwent a balloon-expandable TAV-in-TAV procedure (NYHA III/IV 90% at baseline, 45% at 30 days, and 43% at one year) [14]. Both the mean Kansas City Cardiomyopathy Questionnaire (KCCQ) score improved from 43 at baseline to 76 points at one year, and the mean 6-minute walk test from 164 m at baseline to 248 m at one year (both *p* < 0.0001). Reported quality of life measured by the KCCQ score remained stable in survivors at five-year follow-up [15]. Recent registry data on the SAPIEN 3 valve indicate that TAV-in-SAV provides comparable improvements in functional status and quality of life from baseline to one year, similar to outcomes observed after TAVR for native aortic valve stenosis [16]. Similarly, the CoreValve U.S. Expanded Use Study demonstrated a reduction in NYHA class III/IV from 88% at baseline to 6% at one year, with a mean improvement of 29 points in KCCQ score over the same period in favor of self-expanding TAV-in-SAV (both *p* < 0.0001). Notably, this functional improvement also remained at five years [17].

### 3.3 Limitations of TAV-in-SAV Studies

So far, published studies have consistently shown the safety and efficacy of TAV-in-SAV for failed surgical aortic bioprosthesis, with an early mortality benefit compared to redo SAVR in high-risk patients. The long-term durability and mortality differences between TAV-in-SAV and redo-SAVR remain in favor of redo SAVR if such a procedure is feasible. However, it is important to note that the observational design of published studies is subject to significant limitations, including selection bias, heterogeneity, and residual confounding, even with the use of propensity score adjustment.

Patients undergoing TAV-in-SAV often differed significantly from those receiving redo-SAVR in terms of higher surgical risk, older age, worse functional status, and more comorbidities. Published data were also collected over prolonged periods during which TAVR technology evolved substantially, limiting the validity of direct comparisons with redo SAVR. Some analyses also violate the assumption of proportional hazards, indicating the presence of time-varying treatment effects. Finally, most studies use data from high-volume centers or national registries, which may not reflect outcomes in community-based settings.

## 4. ViV TAVR in Failed Transcatheter Aortic Bioprostheses

### 4.1 Clinical Outcomes and Complications of TAV-in-TAV Studies

Another important consideration in the current literature is the broad application of the term “ViV TAVR”, which is often used to describe both transcatheter aortic valve-in-valve procedures performed within a TAV-in-TAV and those performed within a TAV-in-SAV. However, whether these represent distinct clinical and procedural entities remains an area of active investigation. In the Redo-TAVR registry, Landes *et al*. [18] evaluated outcomes in a propensity score–matched cohort undergoing TAV-in-TAV versus TAV-in-SAV. The authors reported a higher procedural success rate in TAV-in-TAV patients compared to TAV-in-SAV patients (73% vs. 62%, *p* = 0.045). They defined procedural success as a 30-day composite, including freedom from mortality, device-related intervention, major vascular or cardiac complications, a residual mean gradient of less than 20 mmHg, and less than moderate aortic regurgitation. Notably, the authors reported similar 30-day and 1-year mortality rates between TAV-in-TAV and TAV-in-SAV patients (30-day: 3% vs. 4%, *p* = 0.57, and 1-year: 12% vs. 10%, *p* = 0.63). In the TAV-in-TAV group, procedural complication rates were low, with 1.4% of patients having a stroke, 3.3% having valve malposition, 0.9% having coronary obstruction, and 9.6% needing a new permanent pacemaker. No difference was found in procedural safety, defined as a 30-day composite of all-cause mortality, stroke, major bleeding, major vascular complications, coronary obstruction, annular rupture, cardiac tamponade, acute kidney injury, at least moderate aortic regurgitation, need for permanent pacemaker, and device-related intervention (TAV-in-TAV: 70% vs. TAV-in-SAV: 72%, *p* = 0.71).

Similarly, the TRANSIT registry reported a VARC-2 device success rate in TAV-in-TAV patients of 79% with 33% of patients having stenotic degenerated TAV, 56% of them had regurgitant TAV, and 11% of them had a combined degeneration as the indication for ViV TAVR. The authors reported a 30-day mortality rate of 2.9% and a 10% mortality rate at one year. No cases of valve thrombosis or coronary obstruction were recorded [19]. Moreover, the TVT registry of self-expanding TAV-in-TAV procedures reported a VARC-2 device success rate of 95% [20]. Of those, 44% had a stenotic degenerated TAV and 62% had a regurgitant TAV as the reason for ViV TAVR. The 30-day rates of stroke were 3.1%, major vascular complications were 2.1%, major bleeding was 9.1%, and the need for PPM was 5.6%. There were no cases of coronary obstruction or myocardial infarction. The authors reported a 30-day mortality rate of 3.2% and a 17.7% mortality rate at one year. The most recent TVT registry analysis comparing balloon-expandable TAV-in-TAV with native TAVR found comparable outcomes [21]. Coronary obstruction occurred in 0.3%, device embolization in 0.1%, need for a pacemaker in 6.1%, and surgical conversion in 0.5%. No significant differences were observed between groups in 30-day (4.7% vs. 4.0%, *p* = 0.36) or 1-year mortality (17.5% vs. 19.0%, *p* = 0.57), stroke rates at 30 days (2.0% vs. 1.9%, *p* = 0.84) and 1 year (3.2% vs. 3.5%, *p* = 0.80), or device thrombosis (1-year: 0.6% vs. 0.1%, *p* = 0.09).

Although TAV-in-TAV is an attractive alternative to redo SAVR, not all patients can undergo TAV-in-TAV and may require TAVR explant with redo SAVR. In the EXPLANTORREDO-TAVR registry, Tang *et al*. [22] compared the outcomes of patients who underwent TAV-in-TAV with those who underwent TAVR explant with redo SAVR. Patients with TAVR-explant had more preexisting severe PPM (17.1% vs. 0.5%, *p* < 0.001), whereas redo-TAVR patients had more structural valve deterioration as the indication for reintervention (63.7% vs. 51.9%, *p* = 0.023). Compared with TAV-in-TAV, TAVR-explant had higher cumulative mortality at 30 days (13.6% vs. 3.4%, *p* < 0.001) and one-year follow-up (32.4% vs. 15.4%, *p* = 0.001). Using landmark analysis, authors reported that TAVR-explant patients who survived the first 30 days had similar mortality rates to TAV-in-TAV patients at one-year follow-up.

In the EXPLANT TAVR registry, Bapat *et al*. [23] reported similar mortality rates of 13.1% at 30 days and 28.5% at one year in TAVR explant patients. Indications for explanation included endocarditis (43.1%), structural valve deterioration (20.1%), PVL (18.2%), and PPM (10.8%). In the STS database, Fukuhara *et al*. [24] found a 30-day mortality rate of 18% among patients who underwent TAVR explant, with no difference between balloon-expandable and self-expandable devices. However, their findings were notable for higher rates of ascending aortic replacement with self-expandable valve explants compared to balloon-expandable valves (22% vs. 9%, *p* < 0.001), but similar aortic root replacement (19% vs. 24%, *p* = 0.22). In a meta-analysis by Yokoyama *et al*. [25], patients who underwent TAVR explant had a 30-day mortality rate of 16.7% (95% CI: 12.2% to 21.2%), with endocarditis, structural valve deterioration, and PVL as the most common reasons for TAVR explantation.

### 4.2 Patient-Reported Outcomes of TAV-in-TAV Studies

The Redo-TAVR registry showed marked improvement in NYHA class III or IV from 80% at baseline to 10% at 30 days and 13% at 1 year following the TAV-in-TAV procedure [18]. The TRANSIT registry reported similar findings showing improvement in NYHA class III or IV from 74% at baseline to 7% at 30 days and 13% at one year [19]. These results were aligned with findings from the most recent TVT registry of balloon-expandable valves showing sustained improvement both in NYHA class III/IV and KCCQ score from baseline through the first year in patients treated with TAV-in-TAV (NYHA class III/IV: 82% at baseline vs. 14%, KCCQ: mean of 39 at baseline vs. 74 at one year) [21]. The TVT registry of self-expanding valves demonstrated similar improvement in patients’ functional status following TAV-in-TAV procedure (NYHA III/IV class: 62% at baseline vs. 27% at 30 days vs. 25% at one year; KCCQ: mean 38 at baseline vs. 71 at 30 days vs. 75 at one year, *p* < 0.001).

### 4.3 Limitations of TAV-in-TAV Studies

It is important to interpret these findings cautiously, given the inherent limitations of the observational study designs that dominate the current TAV-in-TAV literature. These include small sample sizes, selection bias, patient heterogeneity, and residual confounding, even with the application of propensity score adjustments. It is also important to note that patients undergoing TAVR explant differ significantly from those receiving TAV-in-TAV. They often have prohibitive coronary anatomy, endocarditis, severe PPM, PVL, or require aortic or multivalvular surgery, all of which contribute to an increased risk of mortality.

Nonetheless, current observational data suggest that TAV-in-TAV offers similar short-term safety and mortality outcomes compared to TAV-in-SAV. However, TAV-in-TAV is also not suitable for all patients, and those requiring TAVR explantation with redo SAVR face substantially higher early mortality. Finally, long-term outcomes after TAV-in-TAV are missing and warrant further investigation.

## 5. Four-Step Framework for ViV TAVR

**Fig. 1.**
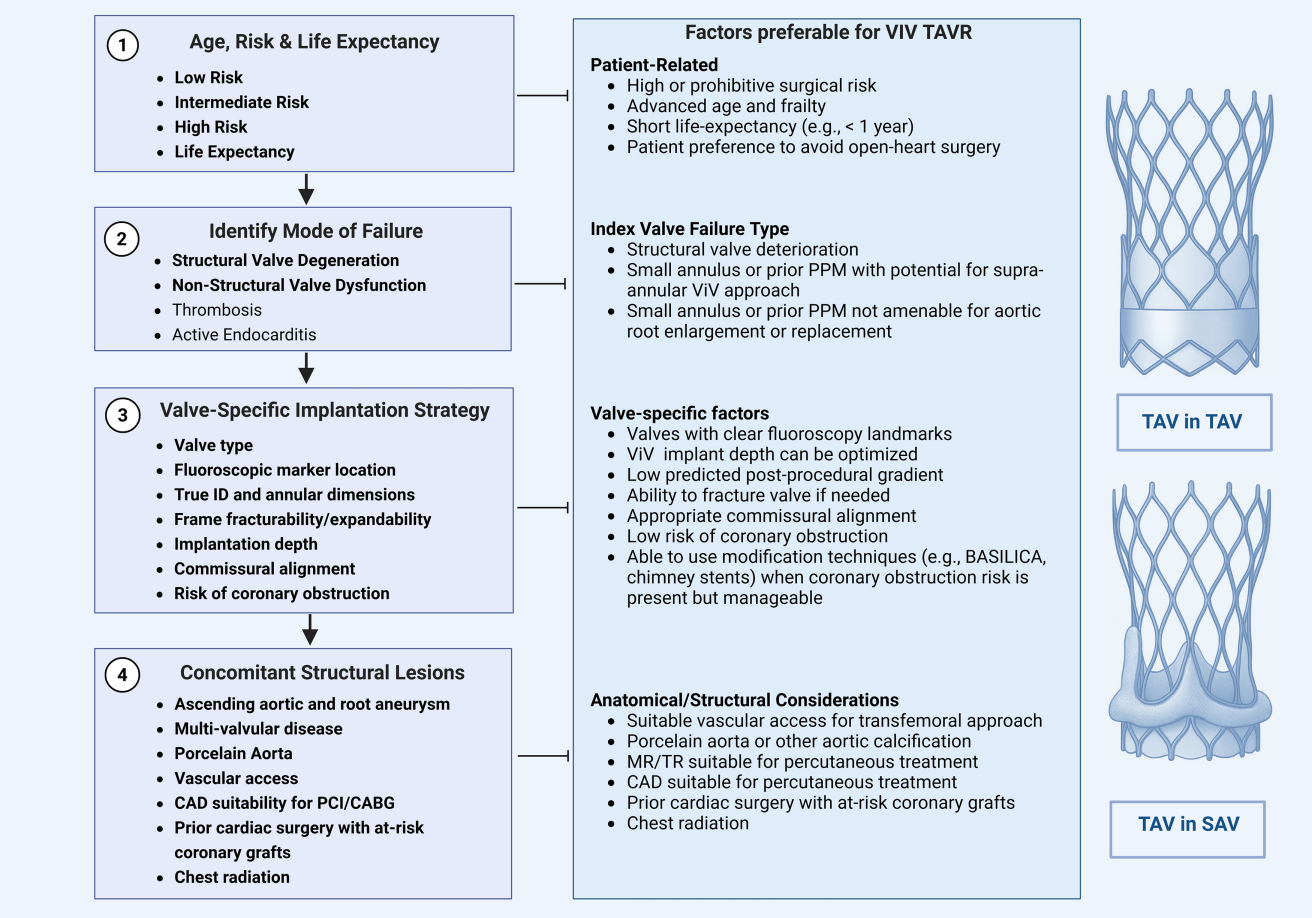
**Stepwise framework for evaluating eligibility for valve-in-valve TAVR**. Evaluation for valve-in-valve transcatheter aortic valve replacement (ViV TAVR) follows a structured four-step approach. Step 1 involves a comprehensive assessment of the patient’s perioperative risk, anticipated life expectancy, and individual preferences to determine candidacy for redo surgical aortic valve replacement (SAVR) versus ViV TAVR. Step 2 determines the mode of failure and assesses whether the failed bioprosthesis has stenosis, regurgitation, or mixed pathology, as each may have distinct procedural implications. Step 3 incorporates valve-specific characteristics that may influence the implantation strategy, such as valve type, fluoroscopic marker location, annular dimensions, true internal diameter, frame fracturability, implantation depth, commissural alignment, and the risk of coronary obstruction. Step 4 focuses on assessing concomitant structural abnormalities, including aortic root or ascending aorta dilatation, valvular regurgitation (mitral/tricuspid), and coronary anatomy, which may impact procedural feasibility and outcomes. TAV-in-SAV, transcatheter aortic valve-in-surigical aortic valve; TAV-in-TAV, transcatheter aortic valve-in-transcatheter aortic valve; MR, mitral regurgitation; TR, tricuspid regurgitation. The figure was created with BioRender.com.

Both American and European guidelines presently do not provide specific guidance on transcatheter valve selection and planning of the ViV procedure. To address this gap, we propose a four-step framework as a pragmatic approach to simplify current evidence into a clinically actionable format and help guide heart team discussions, pre-procedural planning, and patient counseling (Fig. 1).

Step 1 involves a comprehensive assessment of the patient’s perioperative risk, anticipated life expectancy, and individual preferences to determine candidacy for redo SAVR versus ViV TAVR. Step 2 determines the mode of failure and assesses whether the failed bioprosthesis has stenosis, regurgitation, or mixed pathology, as each may have distinct procedural implications. For example, stenotic valves with high transvalvular gradients may require supra-annular valves and pre- or post-dilation. At the same time, regurgitant lesions carry a higher risk of embolization or paravalvular leak, which may necessitate valves with an enhanced sealing skirt or higher radial force. Step 3 incorporates valve-specific characteristics that may influence the implantation strategy, such as valve type, fluoroscopic marker location, annular dimensions and true internal diameter, frame fracturability, implantation depth, commissural alignment, and the risk of coronary obstruction. Step 4 focuses on assessing concomitant structural abnormalities, including aortic root or ascending aorta dilatation, presence of multivalvular disease, and coronary anatomy, all of which may impact procedural feasibility and outcomes.

It is important to note that the applicability of the proposed framework in low-resource settings is limited and variable. The restricted availability of advanced imaging techniques, such as transesophageal echocardiography and cardiac computed tomography (CT), further limits the assessment of valve failure mechanisms and the anatomical feasibility of ViV TAVR. In many centers, procedural planning often relies on simplified approaches, which often limit true shared decision-making, as treatment choices are driven more by availability than by individualized patient assessment. Therefore, this framework is best implemented in high-volume centers with demonstrated expertise in both surgical and transcatheter valve therapies, where ViV TAVR can be performed safely and effectively.

### 5.1 Step 1: Patient’s Surgical Risk, Life-Expectancy, and Patient’s Preference

When selecting between ViV TAVR and redo SAVR for patients with failed bioprosthetic valves, key determinants include patient age, surgical risk, and life expectancy. American and European guidelines emphasize the importance of comprehensive risk assessment using validated tools, such as the STS-PROM and EuroSCORE II, and evaluating frailty, comorbidities, and anatomical feasibility.

ViV TAVR is generally favored in older patients (typically >75–80 years) or those with elevated surgical risk or limited life expectancy, given its minimally invasive nature, reduced perioperative morbidity, and shorter recovery times. In contrast, redo SAVR is preferred in younger patients (typically <65–75 years) with life expectancy longer than one to two years and low surgical risk, due to the superior durability of surgical prostheses and more favorable long-term hemodynamics. For patients in the intermediate-risk category (ages 65–75), either approach may be appropriate, with the final decision informed by patient anatomy, valve characteristics, procedural feasibility, and patient preferences.

Despite growing observational evidence, randomized data directly comparing ViV TAVR and redo SAVR remain limited, underscoring the need for further trials to refine treatment algorithms. Nonetheless, shared decision-making, supported by a multidisciplinary Heart Team, will remain essential to balance short-term procedural safety with long-term valve durability and patient-centered outcomes.

### 5.2 Step 2: Index-Valve Mode of Failure

The long-term management of bioprosthetic valves hinges on an understanding of the mode of their failure. Accurate classification informs procedural planning, guides valve selection, and helps to avoid suboptimal outcomes or unintended complications. The European Association of Percutaneous Cardiovascular Interventions (EAPCI) and, most recently, Valve Academic Research Consortium 3 (VARC-3) describe four potential mechanisms for failure of bioprosthetic aortic valves, including structural valve deterioration (SVD), non-structural valve dysfunction (NSVD), thrombosis, and endocarditis [26, 27]. Key mechanisms of SVD include leaflet disruption, wear and tear, leaflet fibrosis, and calcification. In contrast, NSVD mechanisms include PPM, pannus formation with leaflet entrapment, valve malposition, migration, late embolization, and PVL. Unlike SVD, which usually evolves gradually over time (except in cases of sudden leaflet tears), NSVD often appears at the time of the initial valve replacement and typically remains present during follow-up.

Multiple registries have identified severe PPM, particularly in aortic prostheses ≤21 mm in diameter, as well as PVL, as strong predictors of poor prognosis [28, 29, 30]. Of note, PPM occurs more frequently with surgical than transcatheter aortic valve replacement, likely due to annular size constraints from residual calcification that limit the implantation of larger surgical valves. In contrast, transcatheter heart valves are designed to expand within the native annulus, often accommodating larger or more optimal sizing [29, 31]. Evidence also suggests that in ViV procedures, patients with pre-existing PPM or small surgical bioprostheses achieve lower postprocedural gradients when treated with supra-annular self-expanding valves compared to intra-annular balloon-expandable valves [28, 31, 32]. However, pre-existing severe PPM and PVL may not be adequately addressed with supra-annular ViV alone, particularly in cases where surgical aortic root enlargement or replacement would otherwise be required [22, 23]. In such scenarios, intentional valve fracture using high-pressure balloon inflation may be considered to improve hemodynamics. Importantly, the feasibility and safety of this approach depend on whether the degenerated valve is surgical or transcatheter. While balloon valve fracture is established in treating PPM in failed surgical bioprostheses, intentional overexpansion of transcatheter valves carries increased risks, including acute leaflet dysfunction, annular rupture, and may compromise valve durability [33].

Active infective endocarditis represents a fundamentally different clinical scenario and poses a major contraindication to ViV TAVR. Active endocarditis typically necessitates surgical valve explantation and debridement due to the presence of infection, tissue destruction, and abscess formation, all of which cannot be addressed by transcatheter means. As such, active endocarditis remains an absolute contraindication to ViV TAVR [4]. Nonetheless, in rare and highly selected cases, ViV TAVR has been attempted as a bailout strategy in patients presenting with acute decompensated heart failure due to bioprosthetic valve failure in the setting of treated endocarditis who were deemed prohibitive surgical candidates [34]. Such decisions must be made on a case-by-case basis, ideally through a multidisciplinary heart team discussion, and are not representative of standard practice.

### 5.3 Step 3: Valve-Specific Implantation Strategy

#### 5.3.1 Type of Index-Valve

For ViV planning, it is important to distinguish whether the index surgical valve is stented or stentless. Data from the Valve-in-Valve International Data (VIVID) registry have demonstrated that stentless valves are more prone to regurgitation, whereas stented valves more frequently fail due to stenosis [35]. The VIVID registry has also reported that stentless valves carry a higher risk of malpositioning, embolization, and coronary obstruction. These risks are largely attributable to the absence of fluoroscopic markers to accurately identify the landing zone and the potential for leaflet overdisplacement during ViV deployment. Moreover, subcoronary implanted stentless valves are at a higher risk of coronary obstruction following the ViV procedure due to the proximity of the suture line to the coronary ostia. Notably, the risk of coronary obstruction is not exclusive to stentless valves, as it has also been observed in stented valves with externally mounted leaflets [36]. These include the Abbott Trifecta and Trifecta GT, Dokomis, Crown, and Sorin Mitroflow valves. The external leaflet configuration may increase the likelihood of leaflet displacement towards the coronary ostia during ViV implantation.

#### 5.3.2 Index-Valve Size

Another consideration in ViV planning is recognizing that valves with the same labeled size can have significantly different true internal diameters (ID) depending on the manufacturer and valve design. This variability can contribute to the undersizing of the ViV TAVR valve or the valve underexpansion, leading to PPM and elevated residual gradients. Both PPM and high transvalvular gradients are known predictors of poor clinical prognosis. For example, the true internal diameter of a 21-mm Carpentier-Edwards Perimount valve is roughly equivalent to that of a 23-mm Hancock II bioprosthesis. Such discrepancies highlight the need to select the TAVR valve based on the true ID rather than the labeled size. This assessment should also account for pannus, which can further narrow the effective internal diameter affecting TAVR valve selection and sizing.

#### 5.3.3 Index-Valve Frame Structure

Understanding valve frame structure is also important in ViV planning, particularly when addressing pre-existing PPM and elevated transvalvular gradients. Certain bioprosthetic valves are amenable to intentional valve fracture using high-pressure balloon inflation to enhance post-procedural hemodynamics. Those include, for example, Edwards Magna 3000, Magna Ease 3300, Perimount 2800, Medtronic Mosaic, Mitroflow, Abbott Biocor Epic, Biocor Epic Supra, Epic Plus, Epic Plus Supra, and Dokomis. Other valves, such as the Edwards Inspiris, Carpentier-Edwards Standard and Supra-Annular valves, Perimount 2700, Perceval, and Enable, cannot be fractured but may be partially expanded with balloon dilation. In contrast, valves such as the Medtronic Hancock II, Avalus, Vascutek Aspire, and Abbott Trifecta cannot be fractured or expanded. Recognizing these differences is critical for choosing an optimal ViV strategy and patient outcomes.

#### 5.3.4 Optimal Implantation Depth and Commissural Alignment

Optimal implantation depth is another factor for improving post-ViV hemodynamics by minimizing underexpansion caused by the sewing ring restricting the effective orifice area. Both *in vitro* and clinical studies indicate that more aortic positioning of balloon-expandable and self-expanding valves leads to better hemodynamic outcomes than lower ventricular implantation depths [37]. However, a higher implantation depth can increase the risk of valve embolization and the height of the functional neoskirt. This, in turn, may raise the risk of coronary obstruction, as discussed in more detail below. Besides implantation depth, the commissural alignment of new self-expanding TAVR valves with the commissures of the failed index valve is another factor that can affect new valve hemodynamics and coronary access [38]. This issue is less relevant for low-profile frame TAVR valves because of the ability to access the coronaries above the stent frame [39].

#### 5.3.5 Risk of Coronary Obstruction

**Fig. 2.**
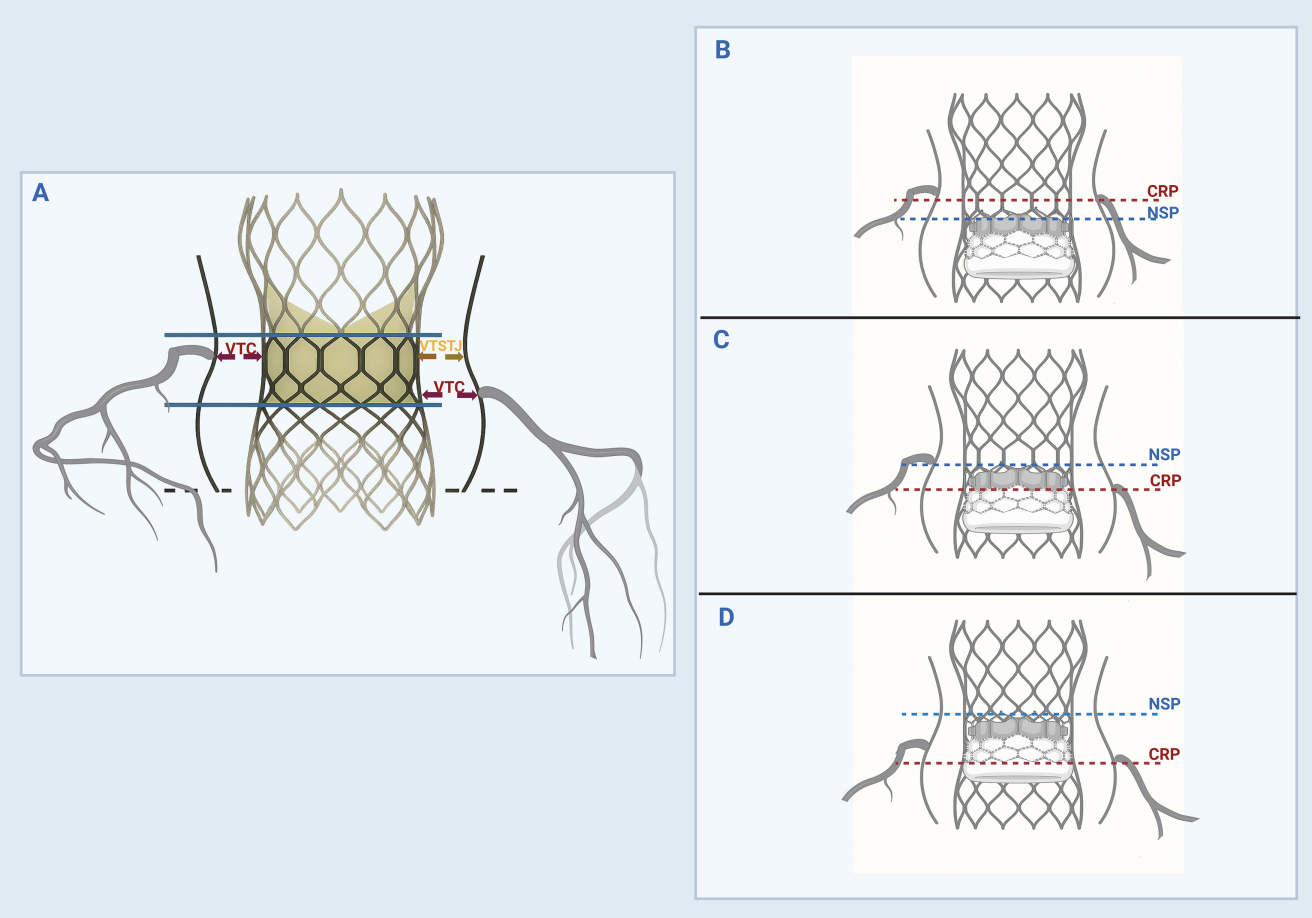
**Identification of ViV anatomy at risk for coronary obstruction and sinus sequestration**. (A) The valve-to-coronary ostial (VTC) and valve-to-sinotubular junction (VTSTJ) distances are calculated by superimposing a virtual ViV TAVR valve within the failed aortic valve and measuring the shortest distance from the outer edge of the virtual valve frame to the coronary ostium and STJ, respectively. The neoskirt height is measured from the inflow (dashed black line) to the pinned leaflet-free edge height of the failed valve (upper solid blue lines). (B) The coronary risk plane (CRP) is a plane below the lowest coronary ostia. The neoskirt plane (NSP) is a plane where the outflow of the ViV TAVR valve pins against the failed index valve. A functional neoskirt is a portion of the neoskirt above the annular plane determined by the implantation depth. If the NSP is below the CRP, then the risk of coronary obstruction is low. (C) If the NSP is above the CRP but below the STJ, then the VTC should be assessed. If the VTC ≥4 mm, then the risk is low. However, if the VTC <4 mm, then the patient is at risk of coronary obstruction and might benefit from an obstruction modification strategy (BASILICA vs. IVUS-guided chimney or snorkel stenting). (D) If the NSP is above CRP and extends above STJ, then the VTC and VTSTJ should be assessed. If either VTC <4 mm or VTSTJ <3 mm then the patient is at risk of coronary obstruction and might benefit from an obstruction modification strategy (BASILICA vs. IVUS-guided chimney or snorkel stenting). The figure was created with BioRender.com.

Pre-procedural CT is an indispensable step for ViV TAVR to identify patients at risk for coronary obstruction and sinus sequestration [40]. In TAV-in-SAV, a virtual valve-to-coronary (VTC) ostia distance of less than 4 mm and a valve-to-sinotubular junction (VTSTJ) distance of less than 3 mm are known to increase the risk of coronary obstruction, especially if the deflected failed valve leaflets extend above a coronary ostium (Fig. 2) [36, 41]. It is important to note that balloon post-dilatation can further reduce these distances because valve overexpansion at the outflow level may result in smaller VTC and VTSTJ distances than initially predicted [42]. This consideration is particularly relevant when balloon valve fracture is anticipated in patients with elevated transvalvular gradients.

In TAV-in-TAV, the main concern is creating a “neoskirt” conduit when the leaflets of the index valve are pushed up against the sinotubular junction (STJ) by the stent frame of the new TAVR valve (Fig. 2) [43]. This situation can cause sequestration of the sinus of Valsalva, which can indirectly obstruct the coronary ostia and impair future coronary access. The ViV with a neoskirt ending below STJ carries a lower risk for coronary obstruction. Notably, the neoskirt height is influenced by the stent frame design and leaflet position. For example, in self-expanding valves, the leaflets can reach the top of the frame, making the neoskirt height equivalent to the frame height. In balloon-expandable valves, leaflets extend about two-thirds up the frame, resulting in a shorter neoskirt height. Implanting short TAV in tall TAV can reduce the neoskirt height, potentially reducing the risk of coronary obstruction [44, 45]. However, it also increases the leaflet overhang of the index valve because it is not fully pinned to the frame, potentially affecting ViV performance and coronary access [46]. For this reason, assessment of multiple CT-derived parameters has been advocated to determine the risk of sinus sequestration and coronary obstruction [47]. These parameters include the coronary risk plane, the neoskirt plane, the functional neoskirt, and the leaflet overhang, as shown in Fig. 2 [47].

Several strategies for risk mitigation of coronary obstruction have been proposed for high-risk ViV candidates. An IVUS-guided chimney or snorkel stenting strategy was described as one in which a separate guidewire and an undeployed stent are placed in the coronary artery, ready to be deployed to protect the coronary ostia if needed [48, 49]. In TAV-in-TAV, chimney stenting is typically not practical because of the risk that the stent may be compressed between the existing and newly implanted valve frames. Alternatively, the BASILICA (Bioprosthetic or Native Aortic Scallop Intentional Laceration to Prevent Iatrogenic Coronary Artery Obstruction) leaflet modification technique and its balloon-assisted variant have recently gained interest. This electrosurgical method involves intentional leaflet laceration to preserve coronary perfusion and has demonstrated feasibility in preventing coronary obstruction during TAVR in native and previously implanted surgical or transcatheter valves [50, 51, 52]. The BASILICA might not be feasible if the commissures between the new and old valve are not aligned [39]. Although early clinical outcomes have been encouraging for both strategies, further bench testing and validation across various TAVR types and sizes are needed to establish broader applicability.

### 5.4 Step 4: Concomitant Structural Lesions

Despite ViV TAVR being an attractive alternative to redo SAVR, certain anatomical and clinical factors often necessitate TAVR-explant instead of ViV. These challenges include low or obstructed coronary ostia, valve malposition or migration, small annulus, annular rupture, risk of mitral valve impingement, or inadequate vascular access, making transcatheter reintervention unfeasible [23]. In contrast, severe calcification of the ascending aorta (“porcelain” aorta), prior chest radiation, and prior cardiac surgery with at-risk coronary grafts would favor TAVR over redo SAVR [4]. The need for concomitant ascending aortic or multi-valvular surgery and coronary artery disease unsuitable for percutaneous treatment are also contraindications to ViV TAVR.

## 6. Conclusions

Aortic ViV TAVR is a viable alternative to redo SAVR for failed surgical or transcatheter bioprosthetic valves. Advances in CT-based preprocedural planning for anatomical assessment, assessing the risk of coronary obstruction, and precise prosthesis sizing and implantation have improved procedural safety and hemodynamic outcomes. Lessons learned from early clinical experience highlight the importance of valve fracture in reducing gradients in small surgical bioprostheses, as well as the use of coronary access modification techniques in preserving coronary perfusion and minimizing the risk of obstruction. Despite these innovations, ViV TAVR remains technically challenging in patients with small valves, significant patient–prosthesis mismatch, paravalvular leak, or high-risk coronary anatomy. To further improve outcomes, simulation-based training, ongoing enhancements in valve design and durability, and well-designed randomized clinical trials are essential to further refine techniques, increase procedural success, and to generate unbiased, long-term outcomes.

## Abbreviations

TAVR, transcatheter aortic valve replacement; ViV, Valve-in-Valve; TAV-in-SAV, transcatheter aortic valve-in-surgical aortic valve; TAV-in-TAV, transcatheter aortic valve–in–transcatheter aortic valve; PPM, patient-prosthesis mismatch; KCCQ, Kansas City Cardiomyopathy Questionnaire; NYHA, New York Heart Association; SVD, structural valve deterioration; NSVD, non-structural valve dysfunction; PVL, paravalvular leak; VTC, valve-to-coronary ostia distance; VTSTJ, valve-to-sinotubular junction distance; STJ, sinotubular junction; CRP, coronary risk plane; NSP, neoskirt plane; SAVR, surgical aortic valve replacement.
